# The effects of consuming a high protein diet (4.4 g/kg/d) on body composition in resistance-trained individuals

**DOI:** 10.1186/1550-2783-11-19

**Published:** 2014-05-12

**Authors:** Jose Antonio, Corey A Peacock, Anya Ellerbroek, Brandon Fromhoff, Tobin Silver

**Affiliations:** 1 Exercise and Sports Sciences, Nova Southeastern University, 3532 S. University Drive, University Park Plaza Suite 3532, Davie, FL 33314, USA

**Keywords:** Protein, Diet, Body composition, Exercise, Supplements

## Abstract

**Background:**

The consumption of dietary protein is important for resistance-trained individuals. It has been posited that intakes of 1.4 to 2.0 g/kg/day are needed for physically active individuals. Thus, the purpose of this investigation was to determine the effects of a very high protein diet (4.4 g/kg/d) on body composition in resistance-trained men and women.

**Methods:**

Thirty healthy resistance-trained individuals participated in this study (mean ± SD; age: 24.1 ± 5.6 yr; height: 171.4 ± 8.8 cm; weight: 73.3 ± 11.5 kg). Subjects were randomly assigned to one of the following groups: Control (CON) or high protein (HP). The CON group was instructed to maintain the same training and dietary habits over the course of the 8 week study. The HP group was instructed to consume 4.4 grams of protein per kg body weight daily. They were also instructed to maintain the same training and dietary habits (e.g. maintain the same fat and carbohydrate intake). Body composition (Bod Pod®), training volume (i.e. volume load), and food intake were determined at baseline and over the 8 week treatment period.

**Results:**

The HP group consumed significantly more protein and calories pre vs post (p < 0.05). Furthermore, the HP group consumed significantly more protein and calories than the CON (p < 0.05). The HP group consumed on average 307 ± 69 grams of protein compared to 138 ± 42 in the CON. When expressed per unit body weight, the HP group consumed 4.4 ± 0.8 g/kg/d of protein versus 1.8 ± 0.4 g/kg/d in the CON. There were no changes in training volume for either group. Moreover, there were no significant changes over time or between groups for body weight, fat mass, fat free mass, or percent body fat.

**Conclusions:**

Consuming 5.5 times the recommended daily allowance of protein has no effect on body composition in resistance-trained individuals who otherwise maintain the same training regimen. This is the first interventional study to demonstrate that consuming a hypercaloric high protein diet does not result in an increase in body fat.

## Introduction

Protein is the most important macronutrient vis-à-vis positive alterations in body composition. Previous work has suggested that protein intakes in the range of 1.2-2.0 grams per kilogram (kg) body weight per day (g/kg/d) are needed in active individuals [1-7]. In contrast, the US recommended daily allowance (RDA) for protein is 0.8 g/kg/d. The average protein intake for US adults is 91 grams daily or ~1.0 g/kg ideal body weight [8]. Thus, the average US adult consumes slightly more than the RDA; however, this level is inadequate for athletes or active individuals who engage in exercise/sport training for several hours per week. Nonetheless, consuming more than the RDA may be considered a ‘high’ intake of protein [9]. In a review by Tipton [10], the definition of a high protein diet may include intakes greater than 15-16% of total energy intake, intakes greater than the RDA or perhaps anything that exceeds 35% of total energy intake. Thus, there is disagreement as to what constitutes a ‘high’ protein diet. We would posit that using percentages as a means of defining ‘low’ or ‘high’ protein intakes is misleading. If one were to consume the hypothetical low calorie diet (ex. 1000 kcal/d), a protein intake of 36% (of total kcals) would be 90 grams; in contrast, it would be 180 grams on a 2000 kcal/d. Thus, it is best to measure protein intake per unit body weight instead of as a percentage of total energy.

According to the Position Stand by the International Society of Sports Nutrition, intakes of 1.4-2.0 g/kg/d are needed for physically active individuals [7]. We would suggest that a ‘high’ protein intake is anything that exceeds 2.0 g/kg/d. However, little is known regarding the effects of protein intake exceeding 2.0 g/kg/d. A recent study compared low, normal and high protein diets [11]. However, even the high protein group was not ‘high.’ They consumed an average of 1.8 g/kg/d of protein. Certainly compared to the sedentary population, 1.8 g/kg/d is ‘high;’ however, 1.8 g/kg/d should be a baseline protein requirement for active individuals.

It is not clear if protein overfeeding will result in body fat gains. Certainly, overfeeding in general will promote body weight and fat mass gain [12]. Furthermore, the composition of meals during times of overfeeding will differentially affect body composition. Two weeks of overfeeding on candy versus peanuts showed that waist circumference increased only in the candy group despite the identical increase in caloric intake [12]. This suggests that overfeeding on sugar results in body fat gains in contrast to consuming a natural food comprised of unprocessed carbohydrate and fat. Furthermore, there may be no difference in overfeeding on fat or carbohydrate in terms of fat storage [13]. Presently, the effects of protein overfeeding in resistance-trained individuals is unknown. Therefore, the purpose of this investigation was to determine the effects of a high protein diet on body composition in resistance-trained men and women in the absence of changes in training volume.

## Methods

### Subjects

Forty resistance-trained subjects volunteered for this investigation. Subjects were unequally randomized to a control (CON n = 10) or high protein diet (HP n = 20) group. The purpose of unequal randomization was to take into account the loss of subjects from potential lack of compliance due to the high protein diet as well as gaining additional information on the treatment itself [14]. Participants were otherwise healthy resistance-trained men and women who had been resistance training regularly for the last 8.9 ± 6.7 years and an average of 8.5 ± 3.3 hours per week. Individuals in the control group were instructed to maintain the same dietary and training habits over the course of the study. On the other hand, the subjects in the high protein diet group were instructed to consume 4.4 grams of protein equal to 4.4 g/kg/d. All procedures involving human subjects were approved by Nova Southeastern University’s Human Subjects Institutional Review Board in accordance with the Helsinki Declaration, and written informed consent was obtained prior to participation.

### Food diary, workout Log, body composition

Subjects kept a daily diary of their food intake via a smartphone app (MyFitnessPal®). The use of mobile apps for diet self-monitoring have been previously used [15]. If they did not use the mobile app, subjects instead kept a paper diary and their daily food intake was measured via the Nutribase® program. In order to maintain a high protein diet, subjects consumed commercially available whey and casein protein powder (MusclePharm® and Adept Nutrition [Europa®]). Otherwise, the rest of their dietary protein was obtained from their normal food intake.

Height was measured using standard anthropometry and total body weight was measured using a calibrated scale. Body composition was assessed by whole body densitometry using air displacement via the Bod Pod® (COSMED USA, Concord, CA). All testing was performed in accordance with the manufacturer’s instructions. Briefly, subjects were tested while wearing only tight fitting clothing (swimsuit or undergarments) and an acrylic swim cap. The subjects wore the exact same clothing for all testing. Thoracic gas volume was estimated for all subjects using a predictive equation integral to the Bod Pod® software. The calculated value for body density used the Siri equation to estimate body composition. Data from the Bod Pod® included body weight, percent body fat, fat free mass and fat mass. All testing was done with each subject at approximately the same time of day. Also, all subjects were required to keep a daily workout log showing the exercises with reps and sets performed. Volume load (repetitions × weight) was measured to ensure subjects did not alter their training regimen.

### Statistical analysis

Data were analyzed utilizing a 2-way Analysis of Variance (ANOVA) with Tukey’s test used for post-hoc analysis. Data are expressed as mean ± SD. A p value of <0.05 was considered significant.

## Results

Forty subjects were initially recruited for this investigation. Ten subjects dropped out. Of the 10, three stated an inability to consume the protein needed for the study and one subject complained of gastrointestinal distress. Six did not provide a reason. Thirty healthy resistance-trained individuals participated in this study (mean ± SD; age: 24.1 ± 5.6 yr; height: 171.4 ± 8.8 cm; weight: 73.3 ± 11.5 kg; 11 female, 29 male). There were no differences between groups for any of the baseline measures (Table 1).

**Table 1:** Subject characteristics

	**Age years**	**Height cm**	**Weight kg**
**Control n** = 10 (2 female, 8 male)	22.0 ± 2.6	174.3 ± 8.2	76.4 ± 9.9
**High Protein n** = 20 (9 female, 11 male)	25.2 ± 6.3	170.0 ± 8.9	71.8 ± 12.2

Data are mean ± SD. There were no significant differences for any of the variables. *cm* centimeters, *kg* kilograms.

There were no statistically significant changes pre vs post or between groups for any of the body composition variables (Table 2).

**Table 2:** Body composition

	**Control**			**HP**		
	**Pre**	**Post**	**Change**	**Pre**	**Post**	**Change**
**BW (kg)**	76.4 ± 9.9	77.2 ± 9.9	0.8 ± 1.6	71.8 ± 12.2	73.5 ± 12.5	1.7 ± 1.9
**FFM (kg)**	65.2 ± 11.7	66.5 ± 11.7	1.3 ± 2.0	59.5 ± 10.9	61.4 ± 11.6	1.9 ± 2.4
**FM (kg)**	11.2 ± 4.7	11.4 ± 5.0	0.3 ± 4.7	12.3 ± 7.0	12.0 ± 6.2	−0.2 ± 2.2
**% BF**	15.1 ± 6.9	14.2 ± 6.9	−0.9 ± 1.7	16.9 ± 8.3	16.3 ± 7.5	−0.6 ± 2.6

Data are mean ± SD. There were no significant differences for any of the variables. *BW* body weight, *FFM* fat free mass, *FM* fat mass,% *BF* percentage body fat, *HP* high protein.

There were no changes in training volume (Table 3). The dietary data are summarized in Table 4. There were no significant changes in the control group for any of the variables. There was a significant increase in total energy and protein intake in the high protein group. It should be noted that every subject in the high protein group consumed protein powder in order to meet the requirements for the study. Otherwise, it would be have virtually impossible or highly unlikely that one could consume a 4.4 g/kg/d via food alone.

**Table 3:** Training volume

	**VL/day**	
	**Pre**	**Post**
**Control**	37148 ± 40979	41847 ± 49022
**HP**	32481 ± 34193	34601 ± 34604

Data are mean ± SD. There were no significant differences for any of the variables. *HP* high protein, *VL* volume load (calculated as reps × weight).

**Table 4:** Dietary intake

	**Control**		**HP**	
	**Pre**	**Post**	**Pre**	**Post**
**Kcal**	2295 ± 639	2052 ± 532	2042 ± 838	*^#+^2835 ± 865
**CHO g**	236 ± 56	227 ± 80	198 ± 100	226 ± 114
**PRO g**	149 ± 49	138 ± 42	162 ± 69	*^#+^307 ± 69
**Fat g**	79 ± 33	77 ± 30	79 ± 34	87 ± 30
	**Control**		**HP**	
	**Pre**	**Post**	**Pre**	**Post**
**Kcal/kg/d**	29.2 ± 6.3	26.2 ± 5.3	28.9 ± 10.8	*^#+^39.9 ± 9.9
**CHO g/kg/d**	3.0 ± 0.7	2.9 ± 0.9	2.8 ± 1.3	3.2 ± 1.6
**PRO g/kg/d**	1.9 ± 0.5	1.8 ± 0.4	2.3 ± 1.0	*^#+^4.4 ± 0.8
**Fat g/kg/d**	1.0 ± 0.4	1.0 ± 0.3	1.1 ± 0.4	1.2 ± 0.4
	**Control**		**HP**	
	**Pre**	**Post**	**Pre**	**Post**
**CHO %**	42.3 ± 8.0	43.1 ± 7.2	36.2 ± 9.9	29.6 ± 8.7
**PRO %**	26.7 ± 4.6	27.8 ± 5.7	30.5 ± 8.7	*^#^45.5 ± 9.9
**Fat %**	31.0 ± 8.5	28.9 ± 5.7	34.2 ± 9.6	27.0 ± 6.9

Data are mean ± SD. P < 0.05 *High Protein Post vs High Protein Pre. ^#^High Protein Post vs Control Post. ^+^High Protein Post vs Control Pre. *CHO* carbohydrate, *PRO* protein, *g* grams, *kg* kilograms, *d* days, *HP* high protein.

## Discussion

The key finding in the present study is that consuming a hypercaloric high protein diet has no effect on body composition in resistance-trained individuals. This is the first investigation in resistance-trained individuals to demonstrate that consuming a high protein hypercaloric diet does not result in a gain in fat mass. On average, they consumed 4.4 g/kg/d of protein which is more than five times the recommended daily allowance [16].

It should be noted that in previous studies, subjects that consumed a hypocaloric diet that is higher in protein and lower in carbohydrate, experienced more favorable alterations in body composition [17-20]. However, the effects of consuming extra calories above normal baseline intake coupled with changes in macronutrient content have not been fully elucidated. The current investigation found no changes in body weight, fat mass, or fat free mass in the high protein diet group. This occurred in spite of the fact that they consumed over 800 calories more per day for eight weeks. The high protein group consumed an extra 145 grams of protein daily (mean intake of 307 grams per day or 4.4 g/kg/d). This is the highest recorded intake of dietary protein in the scientific literature that we are aware of [21-30].

The results of the current investigation do not support the notion that consuming protein in excess of purported needs results in a gain in fat mass. Certainly, this dispels the notion that ‘a calorie is just a calorie.’ That is, protein calories in ‘excess’ of requirements are not metabolized by the body in a manner similar to carbohydrate. Recently, Bray et al. demonstrated that a relatively higher amount of protein does not contribute to an additional gain in fat mass [11]. In this investigation, subjects consumed a diet that exceeded their normal caloric intake by 954 kcal/d. Subjects were randomized into one of three groups: low protein (5% of total energy from protein), normal protein (15%) and high protein (25%). After a treatment period of eight weeks, fat mass increased in all three groups equally (~3.5 kg); however, lean body mass decreased by 0.7 kg in the low protein group in contrast to a gain in the normal (2.9 kg) and high protein (3.2 kg) group. According to the investigators, calories alone contributed to the increase in fat mass; however, protein contributed to gains in lean body mass but not fat mass [11]. Thus, eating extra calories will result in a gain in body fat; however, overfeeding on protein will also result in a gain in lean body mass perhaps due to an increase in muscle protein synthesis.

There are profound differences between the investigation by Bray et al. and the current one. For instance, the current investigation used highly trained subjects whereas the participants in the Bray et al. study did not exercise. What is intriguing is that subjects in the high protein group (Bray et al.) consumed 135 grams of protein daily (~1.8 g/kg/d) compared to their baseline intake of 93 grams (~1.2 g/kg/d). This is less than the amount of protein consumed at baseline for subjects in the current study (~1.9-2.3 g/kg/d). The gain in lean body mass experienced by the subjects in the Bray et al. study suggest that their initial protein intake was inadequate to begin with. Therefore, non-exercising subjects should consume protein at levels twice the recommended daily allowance while keeping carbohydrate and fat intake the same. This dietary strategy alone may promote gains in lean body mass.

On the other hand, the subjects in the current study were resistance-trained subjects who were instructed to not alter their training regimen. Thus, the lack of body composition changes in our group may be attributable to the fact that it is very difficult for trained subjects to gain lean body mass and body weight in general without significant changes in their training program.

An overfeeding study by Tchoukalova et al. demonstrated a gain in fat mass with no change in fat free mass [31]. In this investigation, all subjects consumed a diet that consisted of 50% carbohydrate, 15% protein, and 35% fat. Subjects were instructed to eat until they were ‘more full than usual.’ The extra calories were provided via the choice of an ice cream shake (402 kcal, 40% fat), a king-sized Snickers bar (510 kcal) (Mars Inc.), or Boost Plus (360 kcal/8 oz) (Nestle Nutrition). It is therefore not surprising that eight weeks of overfeeding on food that is largely comprised of carbohydrate would result in a fat mass gain. This is in agreement with other studies [11,12]. Carbohydrate overfeeding has been shown to elevate de novo lipogenesis; moreover, excess carbohydrate may be converted to fat via both hepatic and extrahepatic lipogenesis [13,32].

Norgan et al. had six young men overfeed for 42 days by 6.2 MJ/d (~1490 kcal) [33]. The composition of the overfed meals was 49% carbohydrate, 34% fat, and 17% protein. The mean increase in body weight, body fat and total body water was 6.03, 3.7, and 1.8 kg, respectively. They did not measure body composition per se; however, it would seem reasonable that part of that weight gain would lean body mass. The 17% protein intake in the Norgan et al. study is comparable to the ‘normal’ protein group in the Bray et al. investigation which demonstrated a gain in both fat and lean mass. However, it is in contrast with the current investigation which did not show any significant changes in either parameter.

One might suggest that the high thermic effect of protein may make it difficult to gain body weight during times of overfeeding. It has been shown that the greater the protein content of a meal, the higher the thermic effect [34]. Both young and old individuals experience an increase in resting energy expenditure after a 60 gram protein meal (17-21% increase) [35]. Also, the thermogenic response to a mixed meal (440 kcal of carbohydrate [glucose], fat, and protein) differs between lean and obese subjects [36]. In a study by Swaminathan et al., the thermic effect of fat was lower in obese (−0.9%) versus lean individuals (14.4%). In contrast, there was no difference in the thermic effect of glucose or protein. When subjects consumed a mixed meal, the thermogenic response was significantly less in the obese (12.9%) versus the lean individuals (25.0%) [36]. Another investigation found that the thermic effect of a 750 kcal mixed meal (14% protein, 31.5% fat, and 54.5% carbohydrate) was significantly higher in lean than obese individuals under conditions of rest, exercise and post-exercise conditions. According to the authors, “the results of this study indicate that for men of similar total body weight and BMI, body composition is a significant determinant of postprandial thermogenesis; the responses of obese are significantly blunted compared with those of lean men” [37].

The subjects in our study were lean, resistance-trained young men and women. Their baseline protein intake as ~2.0 g/kg/d. It has been previously demonstrated that a higher protein intake is associated with a more favorable body composition even in the absence of caloric restriction [38]. One might speculate that the thermic effect of consuming large amounts of dietary protein in trained subjects exceeds that of untrained but normal weight individuals.

It is unusual that despite no change in their training volume, the ~800 kcal increase in caloric intake had no effect on body composition. This is the first overfeeding study done on well-trained individuals; thus, one might speculate that their response differs from sedentary individuals. Although there was no significant change in the mean value for body weight, body fat, lean body mass or percent fat, the individual responses were quite varied. This may be due to the fact that other dietary factors were not controlled (e.g. carbohydrate intake). There was a mean increase in carbohydrate intake (~14%) in the high protein group. This was not significant due to the wide variation in intakes. Of the 20 subjects in the high protein group, 9 consumed more carbohydrate whereas 11 decreased or maintained the same intake. It is unclear if consuming protein only during the overfeeding period in the absence of fat or carbohydrate intake alterations would differentially impact body composition; however, we would speculate that protein overfeeding alone would likely have no effect on fat mass while promoting gains in lean body mass concurrent with a heavy resistance training regimen geared towards skeletal muscle hypertrophy.

Another factor that may have played a role in the current investigation is the type of protein consumed in the high protein group. Because of the difficulty in consuming 4.4 grams of protein per kg body weight daily, every subject in the high protein group acquired their additional protein calories primarily from whey protein powder. It has been shown that the thermic effect is greater with whey versus casein or soy protein [39]. Recently scientists demonstrated that consuming similar calories and protein during resistance training in initially untrained individuals resulted in greater gains in lean body mass in the whey supplemented group versus soy or carbohydrate [40]. Another investigation found that muscle protein synthesis after whey consumption was approximately 93% greater than casein and approximately 18% greater than soy. Furthermore, the same pattern held when measured post-exercise (whey > soy > casein) [41]. On the other hand, 48 grams of both whey and rice protein isolate consumed post resistance exercise improved indices of body composition and exercise performance similarly [42]. Thus, one might speculate that if the protein dose or intake is sufficiently high, it may not matter what that particular protein source may be.

## Conclusion

This is the first investigation in resistance-trained individuals which demonstrates that a hypercaloric high protein diet does not contribute to a fat mass gain. Furthermore, there was no change in body weight or lean body mass. This is in contrast with other overfeeding studies which showed gains in body weight, fat mass and lean body mass; however, those investigations were performed in non exercise-trained individuals that were consuming a lower protein diet (in comparison to our study). It should be noted that the subjects in the current study did not alter their training. It would be intriguing to ascertain if a high protein diet concurrent with a heavy resistance bodybuilding training regimen would affect body composition (i.e. increase lean body mass and lower fat mass).

We did not measure blood indices to determine if any side effects (i.e. renal or hepatic function) occurred in the high protein group. A few subjects did complain of gastrointestinal distress as well as feeling ‘hot’ (i.e. their body temperature was chronically elevated). Future research should focus on trained subjects using a single source of protein during overfeeding. Furthermore, a heavy resistance program geared towards skeletal muscle hypertrophy in conjunction with protein overfeeding needs further investigation.

## Competing interests

JA is the CEO of the International Society of Sports Nutrition. The protein powder was provided by MusclePharm® and Adept Nutrition (Europa® Sports Products brand); both are sponsors of the ISSN conferences.

## Authors’ contributions

JA (corresponding author) was responsible for the study design, the statistical analysis and the writing of the manuscript. AE and BF was involved in the execution of the measurements. CP and TS provided assistance in the study design, statistical analysis and editing of the manuscript. All authors read and approved the final manuscript.
